# Diabetic Retinopathy: Animal Models, Therapies, and Perspectives

**DOI:** 10.1155/2016/3789217

**Published:** 2016-01-06

**Authors:** Xue Cai, James F. McGinnis

**Affiliations:** ^1^Department of Ophthalmology, Dean McGee Eye Institute, Oklahoma University Health Sciences Center, Oklahoma City, OK 73104, USA; ^2^Department of Cell Biology, Oklahoma University Health Sciences Center, Oklahoma City, OK 73104, USA; ^3^Oklahoma Center for Neuroscience, Oklahoma University Health Sciences Center, Oklahoma City, OK 73104, USA

## Abstract

Diabetic retinopathy (DR) is one of the major complications of diabetes. Although great efforts have been made to uncover the mechanisms underlying the pathology of DR, the exact causes of DR remain largely unknown. Because of multifactor involvement in DR etiology, currently no effective therapeutic treatments for DR are available. In this paper, we review the pathology of DR, commonly used animal models, and novel therapeutic approaches. Perspectives and future directions for DR treatment are discussed.

## 1. Introduction

Diabetic retinopathy (DR) is one of the major complications of diabetes and is the leading cause of blindness among working people in developed countries. The symptoms are elevated blood sugar levels, blurred vision, dark spots or flashing lights, and sudden loss of vision. The development of DR can be divided into nonproliferative DR (NPDR; subdivided into mild, moderate, and severe stages) with microaneurysms, hard exudates, hemorrhages, and venous abnormalities [1, 2] and proliferative DR (PDR; advanced stage) with neovascularization, preretinal or vitreous hemorrhages, and fibrovascular proliferation [1, 2]. Development of glaucoma, retinal detachment, and vision loss may also happen at this stage. DR may cause macular edema when blood and fluid leak into the retina caused by swelling of the central retina [3]. DR is not easily diagnosed at early stages but is more readily noticed with the advanced stages or with edema. Multiple techniques have been used for detection, diagnosis, and evaluation of this disease including fundoscopic photography, fluorescence angiography, B-scan ultrasonography, and optical coherence tomography (OCT) [4].

## 2. Pathology and Molecular Mechanism of DR

Initially, DR was considered a microvascular complication of endothelial dysfunction, as it is characterized by capillary basement membrane (BM) thickening, pericyte and endothelial cell loss, blood-retinal barrier (BRB) breakdown and leakage, acellular capillaries, and neovascularization [5, 6]. However, it is currently acknowledged that before the typical features of DR occur and can be clinically diagnosed, cellular, molecular, and functional changes are evidenced in the retina [7, 8], where all types of retinal cells are affected including ganglion cells [5, 6, 9]. Also, thinning of the inner nuclear layer (INL), reduction in synapse numbers and synaptic proteins, changes in dendrite morphology, and retinal pigment epithelium (RPE) dysfunction occur in DR and result in the gradual loss of retinal function [9]. In addition, glia activation and innate immunity/sterile inflammation [5, 6] occur early in DR. Therefore, DR is not only a vascular disease but also a neurodegenerative disease.

DR shares numerous similarities in its etiology and pathology with other neovascular diseases which have been documented to be associated with chronic inflammation, including increased vascular permeability, edema, inflammatory cell infiltration, tissue destruction, neovascularization, proinflammatory cytokines, and chemokines in the retina [3, 10]. Some of the potential risk factors leading to the pathology of other neovascular diseases also contribute to the pathology of DR.

Diabetes is the number one risk factor for the development of DR. Type 1 diabetes (juvenile diabetes, in which no insulin is made) is more likely to develop vision loss than type 2 diabetes (adult onset diabetes with insufficient insulin synthesis). In addition, race (Hispanic and African Americans), smoking, hyperglycemia (high blood sugar), hypertension (high blood pressure), and hyperlipidemia (high cholesterol) or dyslipidemia are also high risk factors [11, 12]. Vascular endothelial growth factor (VEGF) elevation induces a decrease in the tight-junction proteins and breakdown of the BRB [13], an increase of leukostasis within retinal vessels [14], inflammation [15, 16], upregulation of ICAM-1 (intercellular adhesion molecule-1) expression, an increase in all NOS (nitric oxide synthase) isoforms [17], and a metabolic imbalance in inorganic phosphate [18], all of which have been reported to contribute to DR pathology. Multiple interconnecting biochemical pathways, including an increased polyol pathway, elevated hexosamine biosynthesis pathway (HBP), activation of protein kinase C (PKC), hemodynamic changes, and advanced glycation end product (AGE) formation [5, 6, 14, 19], have also been found to play key roles in development of DR. RhoA is a small guanosine-5′-triphosphate-binding protein and acts as a GTPase. The RhoA/mDia-1 (mammalian diaphanous homolog-1)/profiling-1 [20] or RhoA/ROCK1 (Rho-associated coiled-coil-containing protein kinase 1) [21] pathways have been shown to be involved in the pathology of DR via triggering microvascular endothelial dysfunction. Activation of these pathways leads to the increase of growth factors such as VEGF and insulin-like growth factor-1 (IGF-1), activation of the renin-angiotensin-aldosterone system (RAAS), subclinical inflammation, and capillary occlusion [14]. Also increased endoplasmic reticulum (ER) stress and oxidative stress [22] resulting from deregulation of ER and mitochondrial quality control by autophagy/mitophagy, RPE dysfunction, genetic variants, and epigenetic changes in chromatin, such as DNA methylation, histone posttranslational modifications affecting gene transcription, and regulation by noncoding RNAs [23–26], have also been shown to be associated with DR. Interestingly, deletion of transforming growth factor-*β* (TGF-*β*) signaling results in undifferentiated pericytes that cause retinal changes in structure and function which mimic those of DR [27]. Loss of other gene functions such as BMP2 (bone morphogenetic protein 2) [28] and Toll-like receptor 4 [29] has been implicated in the pathogenesis of DR. Activation of the P2X7 receptor, a member of ligand-gated membrane ion channels, resulted in the formation of large plasma membrane pores that exacerbate the development of DR through induction of inflammation [30]. Recently, a prooxidant and proapoptotic thioredoxin interacting protein (TXNIP) was shown to be highly upregulated in DR and by high glucose (HG) in retinal cells in culture. TXNIP binds to thioredoxin (Trx) inhibiting its oxidant scavenging and thiol-reducing capacity. Hence, prolonged overexpression of TXNIP causes ROS/RNS stress, mitochondrial dysfunction, inflammation, and premature cell death in DR [31]. Collectively, hyperglycemia-induced vascular dysfunction and subsequent tissue damage have been proposed to act through the following four main pathways [32, 33]: (1) increased polyol pathway flux, in which cytosolic redox imbalance occurs with an increased NADH/NAD^+^ ratio via the sorbitol pathway resulting in a decrease in cytosolic NADPH and cellular functions, (2) increased AGE formation, in which nonenzymatic glycosylation of proteins and production of AGEs alter gene expression and AGEs also induce the synthesis of numerous inflammatory cytokines, (3) activation of PKC via the formation of intracellular diacylglycerol (DAG) and AGEs, which contributes to the generation of ROS which induces VEGF and multiple other growth factors and transcription factors, and (4) increased hexosamine pathway flux, in which fructose-6-phosphate is converted to glucosamine-6-phosphate and finally to uridine diphosphate *N*-acetyl glucosamine. This modification results in changes in gene expression and protein function. However, each of the four major pathways is linked by overproduction of superoxide and increased generation of ROS [33], which provides a common target for potential treatment.

## 3. Animal Models

At present, most animal models of DR are rodents, mice, and rats. Based on the experimental approaches to induce DR, these models can be classified as chemically induced, spontaneous, and genetically created. However, knowledge of the molecular mechanisms underlying the initiation and development of DR is insufficient and largely unknown because there are no reliable and appropriate good animal models of spontaneous diabetes in which phenotypic characteristics exactly mimic the pathogenesis of clinical DR. Although various traditionally used animal models of DR present a number of pathological changes similar to those of human DR, several pathological characteristics of human DR, such as retinal neovascularization, cannot yet be fully mimicked in any existing animal model of DR [34].

### 3.1. Chemically Induced Model

The commonly used streptozotocin (STZ) or alloxan induced DR animal models (rats or mice) exhibit rapid onset of hyperglycemia (3 days after treatment) and some of the symptoms of early DR (type I diabetes), such as loss of retinal pericytes and capillaries, thickening of the vascular basement membrane, vascular occlusion, and increased vascular permeability [3, 34, 35]. However, variability of pathological characteristics, such as loss of retinal capillaries, ganglion cell death, and reduction of retinal function, has been reported among different species and even within the same species [3, 34, 35].

### 3.2. Akita Mice

The Akita (Ins2^Akita+/−^) mouse, a spontaneous diabetes model for early stage of DR (type I diabetes), is caused by a missense mutation in the diabetogenic* Insulin 2* gene (*Ins2*) and is characterized by a rapid onset of hyperglycemia and hypoinsulinemia and marked reduction of insulin secretion by 4 weeks of age [36]. Significant increases in vascular permeability were seen when measured at 12 weeks after hyperglycemia. The thickness of the inner plexiform layer (IPL) and INL in the peripheral region was decreased and the number of ganglion cells was significantly reduced when measured at 22 weeks after hyperglycemia [37]. Recently, Hombrebueno et al. reported that the Akita mice exhibit progressive thinning of the retina and cone loss from 3 months onwards, severe impairment of synaptic connectivity at the outer plexiform layer (OPL), and significant reduction in the number of amacrine and ganglion cells [38, 39]. ER stress associated proteins were upregulated in this mouse model [40]. The transportation of proinsulin from the endoplasmic reticulum (ER) to the Golgi apparatus is blocked, and instead the mutant proinsulin is accumulated in the ER forming complexes with BiP (binding immunoglobulin protein) which are eventually degraded [41].

### 3.3. Kimba Mice

The Kimba mice were generated by microinjection of human VEGF_165_ isoform driven by a photoreceptor-specific promoter (rhodopsin). Pathological changes in the retinal vasculature, focal fluorescein leakage, relatively mild degree, and slow onset of neovascularization were shown at 3-4 weeks of age and stable retinopathy persisted for 3 months, which resembles NPDR and early stage of PDR [1]. A thinner outer nuclear layer (ONL) and INL, severe and extensive outer and inner retinal neovascularization, hemorrhage, retinal detachment [1], microaneurysm, leaky capillaries, capillary dropout [42], leaky blood vessels, and BRB loss [42, 43] were presented in this mouse model. However, the mice overexpressing photoreceptor-specific hVEGF are not on a hyperglycemic background and do not induce choroidal neovascularization [1, 42].

### 3.4. Akimba Mice

The Akimba (Ins2^Akita^VEGF^+/−^) mouse, generated from the Kimba (VEGF^+/−^) (trVEGF029) and the Akita (Ins2^Akita^) mice, is a model for advanced DR [42]. This model retains the parental retinal neovascularization with hyperglycemia and displays the majority of signs of advanced clinical DR including more diffuse vascular leakage (compared to the more focal leakage in Kimba mice) and the BRB disruption, which was linked to decreased expression of endothelial junction proteins, pericyte dropout, and vessel loss [42, 43]. With aging, Akimba mice exhibit enhanced photoreceptor loss, thinning of the retina, more severe and progressive retinal vascular pathology, capillary nonperfusion, much higher prevalence and persistence of edema, and retinal detachment [42]. Plasmalemma vesicle associated protein (PLVAP) is an endothelial cell specific protein which is absent in intact BRB but is significantly increased in Akimba mice (and also in Kimba mice). Therefore PLVAP plays an important role in the regulation of BRB permeability [43].

### 3.5. db/db Mice

The db/db (*lepr* ^*db*^) mouse, a spontaneous diabetic model of type 2 diabetes [44, 45], is caused by a mutation in the leptin receptor gene. It exhibits high glial activation, progressive loss of ganglion cells, and significant reduction of neuroretinal thickness. Significant abnormal retinal function is pronounced at 16 weeks of age. In addition, significantly higher levels of glial fibrillary acidic protein (GFAP, a marker for glial cells) expression, increases in accumulation of glutamate, and downregulation of abundant neurotransmission genes were found at 8 weeks of age [44]. Also, breakdown of the BRB is a hallmark of the db/db mice [46] and RPE dysfunction is concomitant with sustained hyperglycemia [45]. Proteomic analysis of 10-week-old retinas from db/db and wild type mice showed that 98 membrane proteins, out of a total of 844, were significantly differentially abundant in db/db versus wild type mice, in which 80 were downregulated and 18 were upregulated in the db/db retinas [47]. The major proteins decreased are synaptic transmission proteins, especially the vesicular glutamate transporter 1 (VGLUT1) [47], which is responsible for the loading of glutamate into synaptic vesicles and is expressed at the ribbon synapses in the photoreceptors and “ON” bipolar cells [48].

### 3.6. New Animal Models

In recent years, two new animal models were reported. One is a transgenic mouse overexpressing insulin-like growth factor-1 (IGF-1), which develops the most retinal alteration seen in human diabetic eyes on a nonhyperglycemic background [49] and exhibits progressive development of vascular alteration (from NPDR to PDR), increased VEGF level, BRB breakdown, vascular permeability, and glial alteration with age (3 months and older) [49, 50]. Retinal neurodegeneration was seen at 6 months of age with the number of bipolar and ganglion cells reduced and a 40% reduction of ONL and INL thickness was observed in 7.5-month-old mice. Microarray analysis on 4-month-old retinas, with evidence of NPDR and gliosis [50], revealed upregulation of genes associated with retinal stress, gliosis, and angiogenesis. Increased GFAP immunostaining was seen at 1.5 months of age and was maintained throughout the entire life. Activation of ERK signaling was detected at 3 months and was more pronounced at 7.5 months. In addition, expression of oxidative stress markers was increased; in particular a striking upregulation of all three subunits of NADPH oxidase, impaired glutamate recycling, and significantly higher levels of TNF-*α* and MCP-1 were seen at 7.5 months [51]. The other model is the hyperhexosemic marmosets (*Callithrix jacchus*) which, with a 30% galactose- (gal-) rich diet for two years, develops significantly high blood glucose levels, vascular permeability, macular edema, increased number of acellular capillaries, pericyte loss, vascular BM thickening, increased vessel tortuosity in the retinas, and microaneurysms. High-speed spectral domain OCT (SD-OCT) scan reveals significant thickening of the foveal and the juxtafoveal area resulting from intraretinal fluid accumulation. Also there are potential break in the RPE and discontinuous photoreceptor layers in the macular area starting at 15 months of galactose feeding. All these characteristics have striking similarities to the human DR [52].

## 4. Current Therapies

During the nonproliferative stages, treatment is usually not recommended because normal visual function is not disturbed at these stages. However, at the advanced stages, the PDR, treatment has to be undertaken. Traditional approaches for treatment of DR and associated microvasculature and neovascularization include laser treatment, optimizing blood glucose level, and controlling blood pressure. Currently, laser treatment (photocoagulation) to stop the leakage and scattered laser burns to shrink abnormal blood vessels and prevent retinal detachment are effective and are widely employed and are the primary treatment strategy. Surgical treatment to remove the vitreous (vitrectomy) is usually taken for advanced PDR in type I diabetes if persistent vitreous hemorrhage or severe tractional retinal detachment occurs. Intravitreal injection of anti-VEGF (Avastin, Lucentis, and Eylea) and corticosteroids to prevent abnormal blood vessel growth are effective and are also beneficial treatments for PDR [2, 19, 53].

Clinical trial (ClinicalTrials.gov number: NCT01627249) phase III study (660 adults) with intravitreal injection of Aflibercept, Bevacizumab, or Ranibizumab for diabetic macular edema (DME) showed that visual acuity was improved, and Aflibercept is more effective when the initial visual acuity is worse [54]. A five-year clinical trial study reported that intravitreal injection of 0.5 mg Ranibizumab with prompt (124 patients) or deferred (111 patients) focal/grid laser treatment for diabetic macular edema resulted in the maintenance of vision gains obtained by the first year through 5 years in most of the eyes [55]. However, another clinical trial study (322 of 582 eyes) showed that repeated intravitreal Ranibizumab injections for DME may increase the risk of sustained elevation of intraocular pressure or the need for ocular hypotensive treatment [56] and a risk of stroke [2]. Another clinical trial, phase I/II study, evaluating the safety and bioactivity of intravitreal injection of a designed ankyrin repeat protein (MP0112) for specific and high-affinity binding to VEGF in patients with DME, showed reduction of edema and improvement of visual acuity, although several patients showed inflammation [57]. An ongoing clinical trial eliminates the source of inflammation from a new preparation [57].

DR associated pathological factors, molecular signaling pathways, and other mechanisms underlying the pathology of DR, as well as the direct pathological defects (retinal degeneration, synaptic connection impairment and cell loss, accumulation of glutamate, etc.), provide a broad spectrum of potential new therapeutic targets for the treatment of DR. Therapeutic treatment strategies targeting these molecules, components, or defects, including various factors, hyperglycemia- and glutamate-triggered pathways, and microvascular impairment and angiogenesis, have been shown to produce an effective outcome [11, 58, 59]. Chinese traditional medicine HF (He-Ying-Qing-Re formula), in which chlorogenic acid, ferulic acid, and arctin were identified as major components, was shown to have anti-DR effects, although hyperglycemia was not significantly inhibited. Its action on suppression of activation of AGEs and endothelial dysfunction occurs by inactivation of AGEs receptor and their downstream Akt signaling pathway [60]. Deletion of placental growth factor prevents DR by inactivation of Akt and inhibition of the HIF1*α*-VEGF pathway [11, 61]. Recently, angiopoietin-like 4 (ANGPTL 4) was identified as a potential angiogenic factor which was upregulated in the PDR patients and was shown to be independent of VEGF levels and localized in the area of retinal neovascularization. Neutralizing ANGPTL4 antibody can inhibit the angiogenic effect in PDR patients with low VEGF levels or produce an additive effect with anti-VEGF treatment for inhibition of VEGF expression [62].

Preclinical therapies targeting other factors have been reported. A single intravitreal injection of a vector expressing insulin-like growth factor binding protein-3 (IGFBP-3) into diabetic rat retina after 2 months of diabetes restores normal insulin signal transduction via regulation of the insulin receptor/TNF-*α* (tumor necrosis factor-alpha) pathway and leads to the reduction of proapoptotic markers or increases of antiapoptotic markers and the restoration of retinal function [63]. Blockage of TNF-*α* by intravitreal and intraperitoneal delivery of anti-TNF-*α* antibody in STZ-induced mice and Akita mice resulted in a dose-dependent prevention of increased retinal leukostasis, acellular capillary, BRB breakdown, and cell death [64]. Intraperitoneal injection of anti-VEGFR1 antibody (MF1) prevents vascular leakage and inhibits inflammation associated gene expression and abnormal distribution of tight-junction proteins in STZ-induced mice and Akita mice [65].

Fenofibrate is a peroxisome proliferator-activated receptor-*α* (PPAR-*α*) agonist and is known for clinical treatment for dyslipidemia. Recently, it was shown to significantly ameliorate retinal vascular leakage and leukostasis in DR of STZ-induced diabetic rats and Akita mice through downregulation of ICAM-1, MCP-1 (monocyte chemoattractant protein-1), and NF-*κ*B (nuclear factor-kappa B) signaling [66]. Clinical studies demonstrated that Fenofibrate has protective effects on progression of proliferative DR in type 2 diabetic patients [67, 68]. Now, the use of this medication for DR is approved [69].

Omega-3 polyunsaturated fatty acid (*ω*-3PUFA) has been shown to be decreased in STZ-induced diabetic rat retina [70]. *ω*-3PUFA rich diets enhanced glucose homeostasis and preserved retinal function in db/db/mice, but the effect is independent of preservation of retinal vasculature integrity, inflammatory modulation, and retinal neuroprotection [71].

## 5. Novel Potential Therapeutic Targets 

Because of the complicated etiology of DR, drugs such as inhibitors for signaling pathways and growth factors have been shown to be effective for the treatment of DR but have limitations. Currently, intravitreal injection of anti-VEGF and corticosteroids are popular therapeutics, but a high proportion of patients (~40%) do not respond to these therapies [58, 72]. This implies that other factors or pathways, independent of VEGF, are involved in the development of microvasculature and neovascularization. Therefore, there is an urgent need for finding potential target candidates and for the development of new treatment strategies for DR therapy.

Epigenetic chromatin modifications (DNA methylation, histone posttranslational modifications, and regulation by noncoding RNAs), acting on both* cis*- and* trans*-chromatin structural elements, can be regulated by TXNIP [25]. Aberrant epigenetic modifications have been identified in DR and implicated in the progression of DR [25, 26]. MicroRNAs (miRNAs) are a group of noncoding RNA sequences which are short and highly conservative and can posttranscriptionally control gene expression by degradation or repression of target mRNAs. They are implicated in a variety of biological activities including modulation of glucose, angiogenesis, and inflammatory responses, as well as pathogenesis of diabetes and related complications such as DR [10]. However, conflicting data were seen with different miRNAs. It has been shown that retinal miRNA expression was altered in early DR rats induced by STZ, in which miRNAs were differentially regulated compared to the controls without DR [73]. Downregulation of miR-200b has been shown to increase VEGF expression, and polycomb repressive complex 2 (PRC2) (histone methyltransferase complex) represses miR-200b through its histone H3 lysine-27 trimethylation. Thus inhibition of PRC2 through histone methylation of miR-200b increases miR-200b and reduces VEGF in STZ-induced diabetic rats [74]. The 3′-untranslated region (3′-UTR) of mRNA sequence contains regulatory regions including binding sites for miRNAs to repress translation and degrade mRNA transcripts. In DR rats, miRNA-195 was significantly upregulated after one month of diabetes, and the antioxidant enzyme MnSOD level was reduced.* In situ* hybridization indicated that miR-195 was overexpressed in the cells of INL and ONL and ganglion cell layers, but sirtuin 1 (SIRT1) was downregulated. SIRT1 is involved in many biological processes including cell survival and metabolism and miR-195 binds to the 3′-UTR of SIRT1 to regulate its expression. Intravitreal injection of miR-195 antagomir leads to downregulation of SIRT1, thus preventing DR damage caused by SIRT1-mediated downregulation of MnSOD [75]. Collectively, increasing amounts of data demonstrate the active involvement and critical role of miRNAs in development of DR, although the exact mechanisms by which miRNA or miRNAs act are not known. Increased knowledge of how miRNAs function as therapeutic agents will lead to their effective use in the treatment of DR.

Reactive oxygen species (ROS), the primary causative factor for a variety of diseases, have been shown to play an important role in promoting DR [12, 58, 76]. As a treatment target, evidence from preclinical and clinical studies indicates that antioxidant therapies which directly target ROS-producing enzymes are beneficial, although the outcome of large clinical trials has been less promising [76]. However, nuclear factor erythroid 2-related factor 2 (Nrf2), the regulator of phase II enzymes system and the network of cytoprotective genes [77, 78], is still attractive. Its activators have been proven effective in prevention of the development and progression of DR [79]. Here, we specifically point out that nanomedicine attracts more attention in the past several years because it has been beneficial in a variety of medical applications including its promising effects on disease therapy [80, 81]. We have been using cerium oxide nanoparticles (nanoceria) to treat several animal models for ocular diseases and demonstrated their nontoxic and long-lasting effectiveness in delaying retinal degeneration in* tubby* mice [82] and inhibiting retinal and choroidal neovascularization [83]. Due to their unique physicochemical features, nanoceria themselves exhibit superoxide dismutase and catalase activities under redox conditions and can upregulate phase II enzymes [84] and regulate the common antioxidant gene network downstream of Trx [85]. Nanoceria have an atom-comparable size which enables them to freely cross the cellular and nuclear membrane barriers. In addition, they do not need repeat dosing as is required by other antioxidants. Thus one single dose produces sustained protective effects [82–84] which suggests their great potential to be excellent agents for the treatment of DR.

Stem cells emerged as a regenerative therapeutic strategy for treatment of a variety of diseases because they are undifferentiated and retain their stem cell characteristics and possess the potential to differentiate into many different cell types under certain biological conditions [86, 87]. Stem cells have been obtained from multiple sources and have been shown to have a great potential for tissue repair and ocular disease treatment [87, 88]. Human embryonic stem cells (hESCs) can differentiate into more than 99% pure RPE cells and integrate into the host RPE layer and become matured. Phase I/II clinical trials for assessing the tolerability and safety of subretinal transplantation of hESC-derived RPE cells in patients with Stargardt's macular dystrophy (ClinicalTrials.gov number: NCT01345006) and advanced dry AMD (ClinicalTrials.gov number: NCT01344993) have shown that hESCs improve visual acuity [89]. Assessment of their medium- and long-term safety, graft, and survival in patients is ongoing [90]. Mesenchymal stromal cells (MSCs) have been shown to have multiple effects including tissue repair, secretion of neuroprotective growth factors, suppression of host immune response, and lowering glucose levels [91, 92]. Bone marrow derived MSCs have been reported to be differentiated into retinal cells and rescue retinal degeneration in several animal models [91]. Clinical trial phase I assessing their effects on visual acuity in patients with retinitis pigmentosa (RP) (ClinicalTrials.gov number: NCT01068561) has been completed and phase I/II in patients with AMD and Stargardt (ClinicalTrials.gov number: NCT01518127) will be completed in December 2015 (also see review [92]). However, no clinical study of therapeutic effects of MSCs in DR has been reported. Progress has also been made in using several classes of stem cells (EPCs, endothelial progenitor cells; ASCs, adipose stromal cells; PSCs, pluripotent stem cells) to stimulate both neuroregeneration and vascular regeneration in the diabetic retina [92]. EPCs are circulating cells and can be recruited to the sites of vessel damage and tissue ischemia and promote vascular healing and reperfusion [93]. Clinical studies have shown that altered numbers of EPCs were found in patients of type I and type II diabetes with NPDR and PDR, suggesting that EPCs are potential biomarkers for DME and PDR and may be used as therapeutic modalities to treat DR [72]. Preclinical study of STZ-induced diabetic rats receiving a single intravitreal injection of human derived ASCs at two months after diabetes onset showed significant decreases in vascular leakage and apoptotic cells and downregulation of inflammatory gene expression and improved rod b-wave amplitude within one week after injection [94]. Furthermore, mouse ASCs (mASCs) were intravitreally injected into 5-week-old Akimba mice, and the mASCs integrated and associated with retinal microvasculature. Injection of TGF-*β*1-preconditioned mASCs into P9 Akimba pups resulted in a great decrease in capillary dropout areas and avascular areas [95]. These results suggest that regenerative medicine could be a permanent solution for fighting diabetes and associated complications.

Nevertheless, as we previously mentioned, DR has a complicated etiology and involves many factors. Among these causative factors, genetic background seems to contribute most heavily and current approaches for the treatment of DR can only delay the disease progression and do not provide a complete treatment or cure for DR. Correction of the defective gene(s) appears to be potentially the most effective way for DR treatment (see below). In the clinic, the challenge faced is the lack of detection methods for as yet unknown early clinical symptoms which would enable immediate and proper treatment for inhibition of the progression of NPDR to PDR.

## 6. Perspective and Future Direction for DR Treatment 

With wide exploration of the etiology of the diseases using modern molecular techniques, one finds that almost all the diseases are linked with mutation(s) of a specific gene or multiple genes. Current effective gene therapy methods involve gene replacement therapy in which the defective copy of the gene is replaced by the wild type allele to compliment the defect; or knockdown of the defective gene by RNA interference (RNAi) silences the effects of the mutated gene; or introduces a gene to produce a product causing cell apoptosis (http://www.ghr.nlm.nih.gov/handbook). None of the above mentioned strategies can completely eliminate the products or effects of the defective genes indicating that the diseases cannot be completely cured. CRISPR/Cas9-mediated genome editing, which emerged as a new therapeutic strategy for defective gene repairing, has attracted significant attention in recent years. Indeed, at the 2015 annual ARVO (the association for research in vision and ophthalmology) meeting, several laboratories reported their progress in using this approach to correct (or repair) mutant gene sequences from patient-derived induced pluripotent stem cells (iPSCs) for treatment of inherited ocular diseases such as retinitis pigmentosa, AMD, and other retinal diseases [96–99]. Considering the similarity in the pathogenesis of AMD and DR, CRISPR/Cas9-mediated selective engineering of genes associated with DR or angiogenesis is expected to produce positive and effective treatment of DR.
